# A multifactorial intervertebral disc degeneration model: Integrating inflammation, structural disruption, biomechanical parameters, and neural sensitization

**DOI:** 10.1002/btm2.70120

**Published:** 2026-02-01

**Authors:** Marcia Muerner, Junxuan Ma, Rathina V. Balasubramanian, Chencheng Feng, Julia Fernández Pérez, Aleksandr Ovsianikov, Sibylle Grad

**Affiliations:** ^1^ AO Research Institute Davos Davos Switzerland; ^2^ ETH Zurich Zurich Switzerland; ^3^ Research Group 3D Printing and Biofabrication Technische Universität Wien Vienna Austria; ^4^ Austrian Cluster for Tissue Regeneration Vienna Austria

**Keywords:** animal, bioreactors, disease models, intervertebral disc, intervertebral disc degeneration, low Back pain, organ culture techniques

## Abstract

Intervertebral disc (IVD) degeneration (IVDD) is a major cause of low back pain, yet treatment options remain limited. Robust IVDD models are essential for discovering and validating new regenerative treatments. *Ex vivo* whole organ bioreactor cultures using bovine IVDs are a well‐established approach, with various degeneration models developed on this platform. However, most existing models replicate only isolated aspects of IVDD, failing to reflect its complex nature. There is a critical need for *in vitro* models that more accurately simulate the full spectrum of degeneration phenotypes observed in patients. Combining multiple well‐established degeneration models offers a promising strategy. In this study, we investigated the combined effects of enzyme (papain) and cytokine (tumor necrosis factor alpha [TNFα]) based degeneration inducers on bioreactor loaded bovine IVDs. While papain injection led to a 5.5‐fold higher glycosaminoglycan loss and tissue void formation, TNFα induced inflammatory and catabolic changes relevant to IVDD, including significant aggrecanase‐1 (ADAMTS4) upregulation and a 2.65‐fold increase in interleukin 6 release. Both effects were evident when combined, enabling the manifestation of multiple aspects of IVDD in one model. To also explore implications on nociception, primary bovine dorsal root ganglion neurons were cultured and treated with conditioned medium from the induced degenerative IVDs. Nociceptors treated with degenerative medium showed a 1.51‐fold higher proportion of neurons with a response compared to treatment with control IVD medium. By expanding the range of degenerative changes and bridging them to pain‐associated features, this model provides a valuable platform for testing novel regenerative therapies.

**Translational Impact Statement**

Intervertebral disc degeneration is a major cause of low back pain, a condition with a substantial global burden. This work presents a novel bioreactor loaded intervertebral disc organ degeneration model that will serve as a clinically relevant testing platform for the development of regenerative, anti‐inflammatory, and anti‐nociceptive therapies. By replicating the clinical disease environment, our model can reduce the need for extensive preclinical in vivo studies and hence accelerate the clinical translation of new treatments.

## 1 INTRODUCTION

Low back pain is the top cause of disability worldwide, with an estimated 12% of the global population being affected.^1^, ^2^ Intervertebral disc (IVD) degeneration (IVDD) is widely recognized as a leading cause of low back pain.^3^, ^4^, ^5^, ^6^ IVDs are fibrocartilaginous joints in the spine, connecting adjacent vertebrae. The proteoglycan‐rich nucleus pulposus (NP) in the center of the IVD is laterally encompassed by the collagen‐abundant annulus fibrosus (AF).^7^ Cranially and caudally, the cartilaginous endplates connect the IVD to the vertebral bone.^8^

IVDD is commonly linked to aging, but factors like mechanical loading, traumatic injury, nutrient deficiencies, and genetic predisposition can accelerate its progression, leading to a self‐sustaining degenerative cycle.^7^ In IVDD, a catabolic shift of cell phenotype, a decrease in proteoglycan content, reduced IVD hydration, and diminished intradiscal pressure are observed. Furthermore, cartilaginous endplates may calcify, cell number decreases with impaired nutrient exchange, and chronic inflammation arises. Eventually, nerve and vessel ingrowth are observed and are believed to contribute to the development of nociception and discogenic pain.^9^ As degeneration progresses, structural damage such as osteophyte formation, annular tears, and NP herniation can result in complete mechanical failure.^7^, ^9^ Due to the IVD's avascular nature and slow matrix turnover, the regenerative capacity is limited.

Current treatment strategies for IVDD primarily focus on pain management, rather than targeting the underlying pathogenesis.^10^ In severe cases, surgical intervention is indicated, which is associated with inconsistent outcomes and risks of complications.^11^, ^12^, ^13^ Also, surgical treatments generally fail to restore the full mechanical functionality of the diseased IVD. This highlights a critical need for novel, minimally invasive and regenerative treatment strategies. Emerging strategies, such as cell therapies and biomaterials, hold significant potential to address this challenge.^14^, ^15^

Robust IVDD models are essential for screening and validation of new treatment candidates. The use of whole IVDs, cultured *ex vivo* under mechanical load in bioreactors, is a well‐established procedure.^16^ Physiological loading regimes have been developed to keep whole IVDs viable over several weeks. Since human samples are scarce, coccygeal IVDs from bovines are often used. These are closer in size to human IVDs than rodent IVDs and experience similar pressures in vivo (0.1–0.3 MPa).^17^ Bovine IVDs are typically harvested from animals that are slaughtered for meat production and are generally healthy. Therefore, an IVDD‐like phenotype needs to be induced before testing novel treatment strategies.^18^, ^19^, ^20^

A key limitation of current degeneration models is that they mostly rely on a single method of degeneration (e.g., mechanical, enzymatic, inflammatory), resulting in the induction of only a subset of the complex IVDD phenotype. For instance, void formation is induced by enzymatic treatment such as papain injection or by nucleotomy.^21^, ^22^, ^23^ Alternatively, cytokine injections are employed when the aim is testing anti‐inflammatory agents.^24^, ^25^, ^26^ However, cytokine concentration and timing of administration vary largely among studies and lack standardization. Furthermore, the mechanisms of IVDD and investigated regenerative treatments (e.g., cell therapies) are multifactorial. For example, cell‐based therapeutics are not only designed to alleviate inflammation but may also repopulate the IVD and produce extracellular matrix (ECM) for tissue regeneration.^27^, ^28^, ^29^ Therefore, current models, relying on only a fraction of mechanisms, may omit important insights into the treatment effect. Combining known degenerative inducers is a strategy to recapitulate the complexity of IVDD that so far remains underexplored. Especially, the combination of cytokine and enzymatic disease inducers has not been studied comprehensively.

While current models offer valuable insights into IVD structure and biology, they lack a neuronal component, limiting the study of IVD–neuronal crosstalk, a field of growing interest. Increasing evidence, for instance, suggests that cytokines like tumor necrosis factor alpha (TNFα) or interleukin 1β (IL1β) can drive the expression of neurotrophic factors (e.g., brain‐derived neurotrophic factor and glial cell derived neurotrophic factor).^30^, ^31^, ^32^ Therefore, studying the neuron response and plasticity is necessary to understand the relationship between IVDD and the nociceptive system. The dorsal root ganglion (DRG) is a neuron hub which lies in anatomical proximity to the IVD and contains the first order of nociceptive neurons that encode nociception‐associated signals from the IVD.^33^ Recently, a protocol has been developed to isolate and culture primary DRG cells from freshly slaughtered bovine animals.^34^ This opens the possibility of studying the effects of released factors from the IVD on cultured DRG neurons, enabling investigation of IVD–DRG crosstalk and the mechanisms of IVD‐derived signaling (e.g., through cytokines or ECM components).

To address this lack of models that integrate structural, inflammatory, and nociceptive components, in this work, we study the combined effect of the enzyme papain and the cytokine TNFα—both previously used individually as IVDD inducers.^23^, ^25^ Papain injection has been shown to create structural damage, leading to a void in the NP, enabling the injection of larger biomaterial/cell therapy volumes.^23^ TNFα has been shown to play an important role in inducing proinflammatory and catabolic processes during IVDD pathogenesis.^25^, ^35^, ^36^ By supplementing TNFα to papain‐treated IVDs, we hypothesized to induce a catabolic and inflammatory cellular phenotype, complementing the enzymatic tissue breakdown. To investigate potential changes in nociceptive potential in this combinatory degeneration model, primary bovine DRGs were treated with conditioned medium (CM) from degenerated IVDs.

Overall, this work aimed (1) to assess the single and combined effects of enzyme and cytokine treatments on structural, mechanical, and phenotypic markers of IVDD and (2) to evaluate the potential of DRG sensitization as an *in vitro* readout for nociceptive components.

## 2 MATERIALS AND METHODS

### 2.1 Bovine IVD explants and treatment with papain and TNFα

**FIGURE 1:**
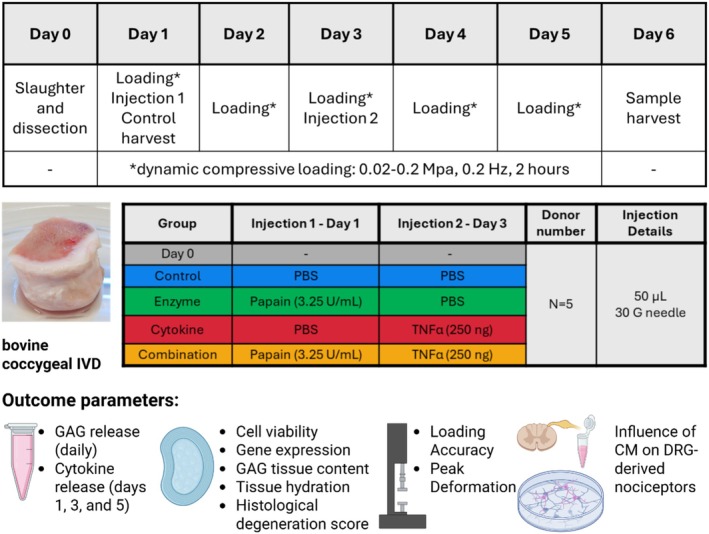
Complete experimental setup for a combinatory *ex vivo* model using an enzymatic and a cytokine agent.

A summary of the experimental setup and group allocations is shown in Figure 1. Briefly, this study included five experimental groups: an enzyme group, where IVDs were injected with 50 μL of 3.25 U/mL papain (from Carica papaya; Roche, Switzerland) on day 1 and 50 μL PBS on day 3; a cytokine group, which received PBS on day 1 and an intradiscal TNFα injection (50 μL, 250 ng, recombinant bovine TNFα; Thermo Fisher Scientific, USA) on day 3; a combination group, which received both treatments; a control group, where only PBS was administered; and day 0 controls. All intradiscal injections were performed using 30G insulin needles. Papain and TNFα were administered on separate days based on preliminary tests showing interference of papain and TNFα (see Data S1, Supporting Information). The TNFα injection was performed on day 3 based on pilot experiments, which showed that TNFα‐induced IL‐6 and IL‐8 release peaked 2–3 days post‐injection and returned close to baseline by day 5 (Data S7). Papain activity (3.25 U/mL) was selected based on literature data and pilot studies, confirming reproducible induction of features of mild–moderate degeneration.^37^ The TNFα dose (250 ng/IVD) was chosen according to previous studies, where 100 ng/cm^3^ IVD volume was shown to be effective, and considering our IVD volumes of 2–3.5 cm^3^.^25^, ^38^

Fresh coccygeal bovine IVDs (diameter 18.02 ± 1.57 mm; height 11.74 ± 1.59 mm; aged 350 ± 223 days; three female, two male animals) were collected and cultured as previously reported.^23^ Briefly, tails were rinsed with Lifo‐Scrub® (B.Braun, Germany) and immersed in 1% betadine solution (Mundipharma, Germany) for 10 min. Soft tissue was carefully removed from the caudal spine. Bony processes were removed using bone pliers. Two parallel cuts were made through the growth plates at both ends of the IVDs using an Exakt 300 Band Saw (Exakt, Germany). IVDs were cleaned using the Pulsavac (Zimmer Biomet, USA) wound debridement system for 30 s, washed in a 10% penicillin–streptomycin (Pen‐Strep; Gibco, Life Technologies, USA) solution for 12 min, followed by a 2‐min rinse in a 1% Pen‐Strep solution. All IVDs were randomly assigned to one of five experimental groups (*n* = 5, each donor represented once in each group).

All IVDs except the day 0 controls were loaded physiologically in a uniaxial bioreactor (dynamic compression 0.02–0.2 MPa, 0.2 Hz, 2 h/d) for five consecutive days. Between loading periods, IVDs were kept free swelling at 37°C and 20% O_2_. Culture medium (DMEM HG supplemented with sodium bicarbonate, pyruvate, 1% Pen‐Strep, 2% fetal bovine serum [FBS], 1% ITS+, 1% nonessential amino acids, 25 mmol/L HEPES [Gibco, Life Technologies, USA], 50 μg/mL ascorbate‐2‐phosphate, and 50 μg/mL primocin [InvitroGen, USA]) was exchanged daily before and after loading.

IVD height changes were measured using calipers daily before and after loading. IVD height after loading was defined as the measured height after each loading relative to the initial IVD height in percentage. IVD height after free swelling was defined as the measured IVD height after free swelling relative to the initial IVD height in percentage.

IVD CM was collected daily before and after loading. The medium was analyzed for sulfated glycosaminoglycans (GAG) using the dimethylmethylene blue (DMMB) method.^39^ On days 1, 3, and 5, IL6 (R&D Systems, USA) and IL8 (Kingfisher Biotech, USA) release into the medium was quantified with bovine‐specific enzyme‐linked immunosorbent assays (ELISA) following the manufacturer's instructions. The release of GAG, IL6, and IL8 per day was normalized to the IVD volume, calculated from initial IVD diameter and height.

On day 6 (respectively day 1 for day 0 controls), tissue was harvested for GAG content, hydration, histology, and gene expression analysis. Briefly, the remaining bony endplate was removed using a scalpel, and the IVD was cut sagittally with a Padget tool. A quarter of the IVD was fixed in 4% formalin (Formafix, Switzerland) for 3 days and embedded in paraffin; 5 μm sections were cut with a microtome and stained with Safranin O/Fast Green. Another quarter was used to harvest ~50 mg tissue from the inner AF (iAF), outer AF (oAF), and NP. Wet weight was noted. Tissues were lyophilized overnight, and further processed for GAG and hydration analysis. Both remaining IVD quarters were snap‐frozen using liquid nitrogen in optimal cutting temperature compound (OCT; Tissue‐Tek, Switzerland); 10 μm slices were cut with a cryostat and stained for lactate dehydrogenase and ethidium homodimer (LDH/ETH) to assess cell viability. Additional sections were collected and stored at −70°C for subsequent RNA isolation for gene expression analysis.

### 2.2 Tissue hydration

After lyophilization the tissue was weighed, and hydration was calculated according to the formula:

$$\text{Tissue hydration}\;\left(\%\right)=100*\frac{{\text{Weight}}_{\mathrm{wet}}-{\text{Weight}}_{\mathrm{dry}}}{{\text{Weight}}_{\mathrm{wet}}}.$$

To assess tissue GAG content, the lyophilized tissue was digested using Proteinase K (0.5 mg/mL in phosphate buffer; Roche, Switzerland) at 56°C until no visible tissue remained, and GAG was measured with the DMMB assay.

### 2.3 LDH/ETH staining

Cell viability was quantified using LDH/ETH staining.^40^, ^41^ Briefly, sections were stained with 1 μg/mL ETH (EtH‐1, Sigma‐Aldrich, USA) for 45 min in a dark, humid chamber. After PBS washes, LDH solution (Polypep, 60 mM lactic acid, 2.64 mM β‐nicotinamide adenine, and 3.67 mM nitroblue powder, pH = 8) was applied and incubated at 37°C in a dark humid chamber for 3 h. Sections were washed, fixed in 4% formalin and coverslipped. Images of the whole section were taken using 10× optics with transmitted (LDH) and fluorescent (ETH) light using an Axioscan7 slide scanner (Zeiss, Germany). A custom‐made ImageJ macro was used to count the cells (correlation coefficient *r* ^2^ = 0.8984 between manual counting and macro, see Data S2). For each IVD region a total area of 5.76 mm^2^ was analyzed. Cells positive for LDH were classified as viable, while cells positive for ETH were classified as dead. Cells positive for both were categorized as viable. Final cell viability was defined as the proportion of viable cells relative to the total cell number.

### 2.4 Safranin O/Fast Green staining and structural scoring

Paraffin sections were deparaffinized, hydrated, and stained with Weigert's Hematoxylin for 10 min to highlight nuclear structures. The slides were then blued in tap water and stained with 0.02% Fast Green in distilled water containing 1 mM glacial acetic acid, and differentiation was performed using 1% acetic acid for 20 s. Subsequently, the sections were stained with 0.1% Safranin O for 12 min, followed by an ethanol differentiation step. Finally, the slides were mounted with Eukitt for visualization. Brightfield images with a magnification of 20× were taken of whole sections using an Axioscan7 slide scanner. Images were evaluated by two independent, blinded researchers using a previously reported scoring system.^23^, ^42^ The final score was determined using the average of the two individual scores, whereby consensus was reached in case of major disagreement. Details about the scoring can be found in Table 1.

**TABLE 1:** Scoring system for Safranin O/Fast Green‐stained histological slides as previously reported.^23^, ^42^

Category	Score	Characteristics
NP matrix staining	0	Proteoglycan staining dominant
	1	Slight reduction in proteoglycan (fading)
	2	Severe reduction in proteoglycan
	3	Loss of proteoglycan staining
AF morphology	0	Well‐organized, well‐defined, uniform collagen lamellae form concentric half‐rings throughout the entire AF
	1	Mild disorganization/delamination of collagen fiber lamellae with some disruption or loss of concentric layers (<25%)
	2	Moderate disorganization/delamination of collagen fiber lamellae with progressive disruption or loss of concentric layers (25%–75%)
	3	Complete disorganization/delamination/collapse of AF; almost all concentric collagen lamellae are severely disrupted or lost (>75%)
Distinction between NP and AF	0	Clear distinction between AF and NP tissue with intense purple proteoglycan ECM staining in NP
	1	Distinction less clear; loss of annular‐nuclear demarcation
	2	Distinction poor; loss of annular‐nuclear demarcation
	3	No discernable annular‐nuclear demarcation

*Note*: All scores range from 0 to 3 (least to most degenerated).

### 2.5 Gene expression analysis

For RNA isolation, cryosections were homogenized in TRI reagent (MRC, USA). Subsequently, the RNeasy Plus Micro Kit (Qiagen, Germany) was used to isolate RNA following instructions provided by the manufacturer. Reverse transcription and real‐time quantitative polymerase chain reaction (RT‐qPCR) were performed using VILO SuperScript reagents (Invitrogen, Thermo Fisher Scientific, USA) and Taqman universal master mix (Applied Biosystems, Thermo Fisher Scientific, USA) with the QuantStudio7 Flex PCR system (Thermo Fisher Scientific, USA).^25^ Genes and primer sequences used are reported in Data S3. Relative quantification was performed using the comparative Ct‐method (2^−ΔΔCt^) with ribosomal protein lateral stalk subunit P0 (RPLP0) as the endogenous reference gene and the control group (PBS injection) as the internal control.^43^

### 2.6 Assessment of loading accuracy and change in peak deformation

Displacement and force values were recorded at a frequency of 10 Hz throughout each loading. Custom‐made Python scripts were used to analyze force and displacement data. To assess loading accuracy, the mean absolute error at the minimum and maximum amplitude of each loading cycle was calculated for each sample and day. Peak deformation per cycle was determined as deformation at maximum compressive loading for each loading cycle. Change in peak deformation was calculated as the peak deformation at the last cycle minus peak deformation at the first cycle measured in millimeter.

### 2.7 DRG neuron sensitization assay

Cells were isolated from fresh bovine DRGs and cultured as described previously (*n* = 4 donors; aged 189 ± 66 days; 3 females, 1 male).^34^ In brief, isolated DRGs were sliced and enzymatically digested with 4 mg/mL collagenase P (Roche, Germany) for 2 h. The digest was passed through a 100 μm cell stainer (Falcon, USA), and density gradient centrifugation was performed on 5 mL of 12% BSA in a 50 mL Falcon tube, which was centrifuged at 1800 rpm for 7 min. The cell pellet was resuspended in DRG culture medium (50% v/v DMEM [Gibco, UK] and F‐12 [Sigma, UK], supplemented with 10% FBS, 20 mM HEPES, 1% Pen‐Strep, and 1% ITS+). Cells were seeded in 18‐well chambers (ibidi, Germany) precoated with poly‐D‐lysine and laminin (both from Sigma, Netherland) (seeding density: 5000 neurons/cm^2^). Two days after cell seeding, IVD CM from the control and combination group (40 μL/well) were used to stimulate the DRG cells for 48 h. TNFα content of CM used for the DRG sensitization study was determined using ELISA (R&D Systems, USA) following manufacturer's instructions.

Calcium imaging was performed as previously described.^44^ DRG cells were loaded with 5 μM Fluo‐4 (ThermoFisher, USA) and imaged in Krebs–Ringer's solution (NaCl 119 mM, KCl 2.5 mM, NaH_2_PO_4_ 1.0 mM, CaCl_2_ 2.5 mM, MgCl_2_ 1.3 mM, HEPES 20 mM and D‐glucose 11.0 mM). Time‐lapse images were taken using a LSM800 confocal microscope (Zeiss, Germany) with 10× optics (excitation wavelength: 488 nm; emission wavelength: 509 nm). At a fixed time point, capsaicin (final concentration 100 nM) was added to the cells to assess the fluorescent spikes as the response of neurons. Subsequently, potassium chloride (KCl, final concentration 50 mM) was added to the DRG cells to identify viable neurons. Only cells exhibiting immediate KCl induced depolarization were considered viable neurons. After calcium imaging, cells were fixed in 4% formalin and stained for calcitonin gene‐related peptide (1:200; anti‐CGRP; Immunostar, USA), neurofilament (1:100; anti‐NF; Thermo‐Scientific, USA) and Hoechst (Sigma, USA).^44^ Cells were imaged using a 10× objective on a LSM800 confocal microscope. Image analysis for calcium imaging and immunofluorescence images was performed with ImageJ and Zen (blue edition, Zeiss, Germany). Calcium spikes were measured using the “multi‐measure” function in ImageJ and defined as snap fluorescence increase followed by a decay. The threshold of peak height was defined using dead neuron fluorescent oscillations. Only spikes higher than the threshold were regarded as biologically relevant calcium signals for analysis. The calcium imaging data were analyzed using an algorithm designed in “R”. To assess group differences in the number of neurons with a high spike, the dataset was categorized based on a threshold. The threshold was set at 0.25, as this value appeared suitable for distinguishing between “low” and “high” peaks. To assess the number of viable cells, the cells responding to KCl addition during calcium imaging were manually counted. For analysis of immunofluorescence images, NF+ and CGRP+ cells were manually counted for each well.

### 2.8 Statistical analysis

Statistical analysis was performed in GraphPad Prism 10 (version 10.1.2, GraphPad Software, United States). For datasets with one independent variable, homogeneity of variance was assumed, and normality was tested using a Shapiro–Wilk test. If normality was confirmed, a *T* test (two groups) or a one‐way ANOVA with Tukey's multiple comparisons test with a single pooled variance (>2 groups) was performed. For normally distributed gene expression data additionally a Bartlett's test was performed to assess significant differences to the control group. If the dataset was not normally distributed, a Mann–Whitney U test (two groups) or a Kruskal–Wallis test with Dunn's multiple comparisons (>2 groups) was performed. For datasets with two independent variables (experimental groups and days of loading) homogeneity of variances was assessed with a homoscedasticity plot and if necessary, data was log‐transformed. Geisser–greenhouse's epsilon was used to correct for violations of sphericity if required. Normality was assumed and a repeated measure two‐way ANOVA with Tukey's multiple comparison test with individual variances was used to detect statistical significance. For neuron sensitization (calcium imaging), classified data was compared using a chi‐square test. Data in figures show mean values and standard deviation if not stated differently in figure caption. *p*‐values <0.05 were considered as significant and annotated with asterisk if not stated differently in figure captions (*p < 0.05, **p < 0.01, ***p < 0.001, ****p < 0.0001).

## 3 RESULTS

### 3.1 Void formation and cell viability

**FIGURE 2:**
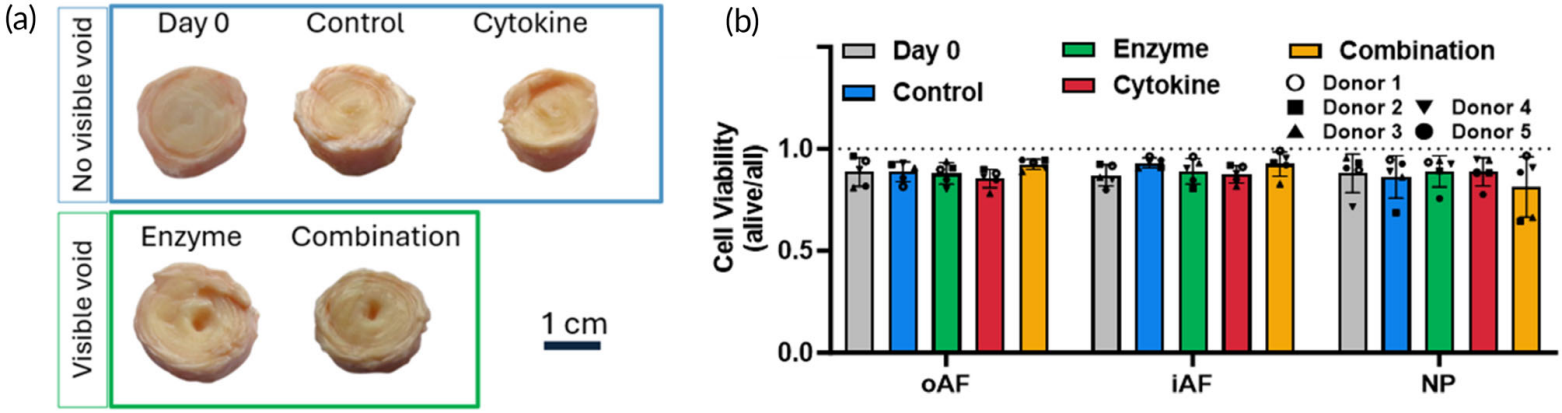
(a) Images taken on the day of harvesting, after the vertebral bone was removed on one side. Clear void formation was only visible when enzyme treatment was performed. (b) Cell viability (alive cells/all cells), *n* = 5. No significant differences between the groups were detected with a Kruskal–Wallis test with Dunn's multiple comparisons test.

Void formation in NP was observed after administration of papain, but not in the control and cytokine groups without papain (Figure 2a). In all experimental groups, cell viability remained >80% for NP, iAF, and oAF with no significant differences between the experimental groups (Figure 2b; Data S4 for representative images).

### 3.2 GAG release into medium, GAG tissue content and tissue hydration

**FIGURE 3:**
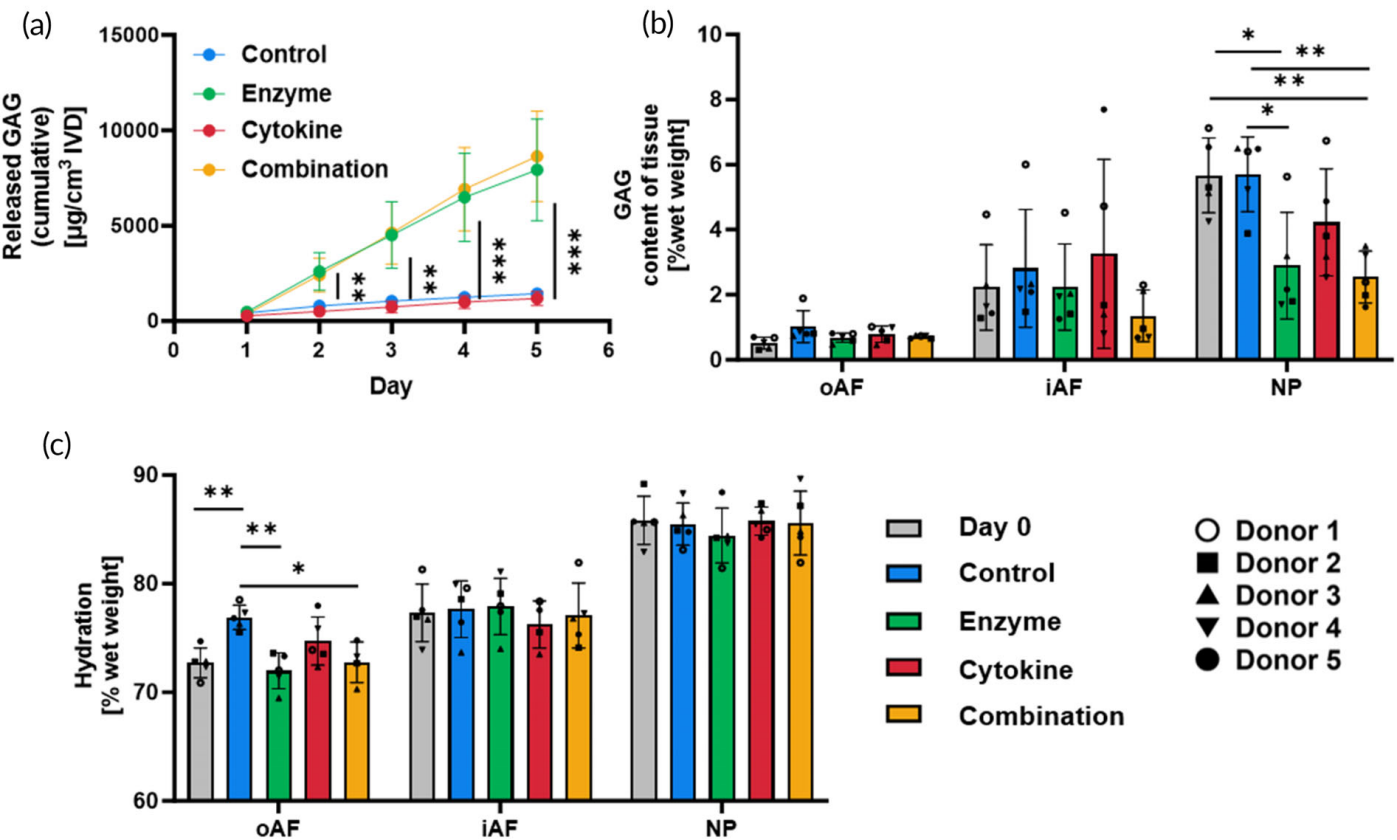
GAG release, GAG content, and IVD hydration. (a) Cumulative GAG release into the medium for all loading days, *n* = 5 IVDs; statistically compared using a repeated measure two‐way ANOVA with Geisser Greenhouse correction and Tukey's multiple comparisons test. A graph showing individual datapoints can be found in Data S5. (b) GAG content as percentage of tissue wet weight, *n* = 5 IVDs; statistically comparing groups within the same tissue using one‐way ANOVA with Tukey's multiple comparisons test or Kruskal–Wallis test with Dunn's multiple comparisons test. (c) Hydration as percentage of tissue wet weight, *n* = 5 IVDs. One datapoint is missing (oAF, combination group) due to a failed measurement. Groups within the same tissue were compared using one‐way ANOVA with Tukey's multiple comparisons test. For all datasets *p*‐values <0.05 were considered as significant (*p < 0.05, **p < 0.01, ***p < 0.001).

Total cumulative GAG release into the medium was increased 5.5‐ and 6.0‐fold for the enzyme and combination group respectively compared to the control group (Figure 3a). Statistical difference between these two groups and the control group was already apparent 1 day after enzyme treatment with 3.3 and 3.0‐fold increases for the enzyme and combination group, respectively. For the cytokine and the control group, total GAG release into the medium remained below 1500 μg/cm^3^ IVD.

Tissue GAG content was not statistically different between the groups for the oAF and iAF (Figure 3b). When papain was administered, NP GAG content was reduced by 51% compared to both day 0 and the control group. The cytokine group fell in between the papain injected and the control groups, with 74% of tissue GAG content compared to day 0 controls. Surprisingly, hydration was not significantly different among the groups in iAF and NP (Figure 3c). For the oAF, tissue hydration was significantly higher in the control group compared to day 0 and the two papain injected groups.

### 3.3 Histological staining and scoring

**FIGURE 4:**
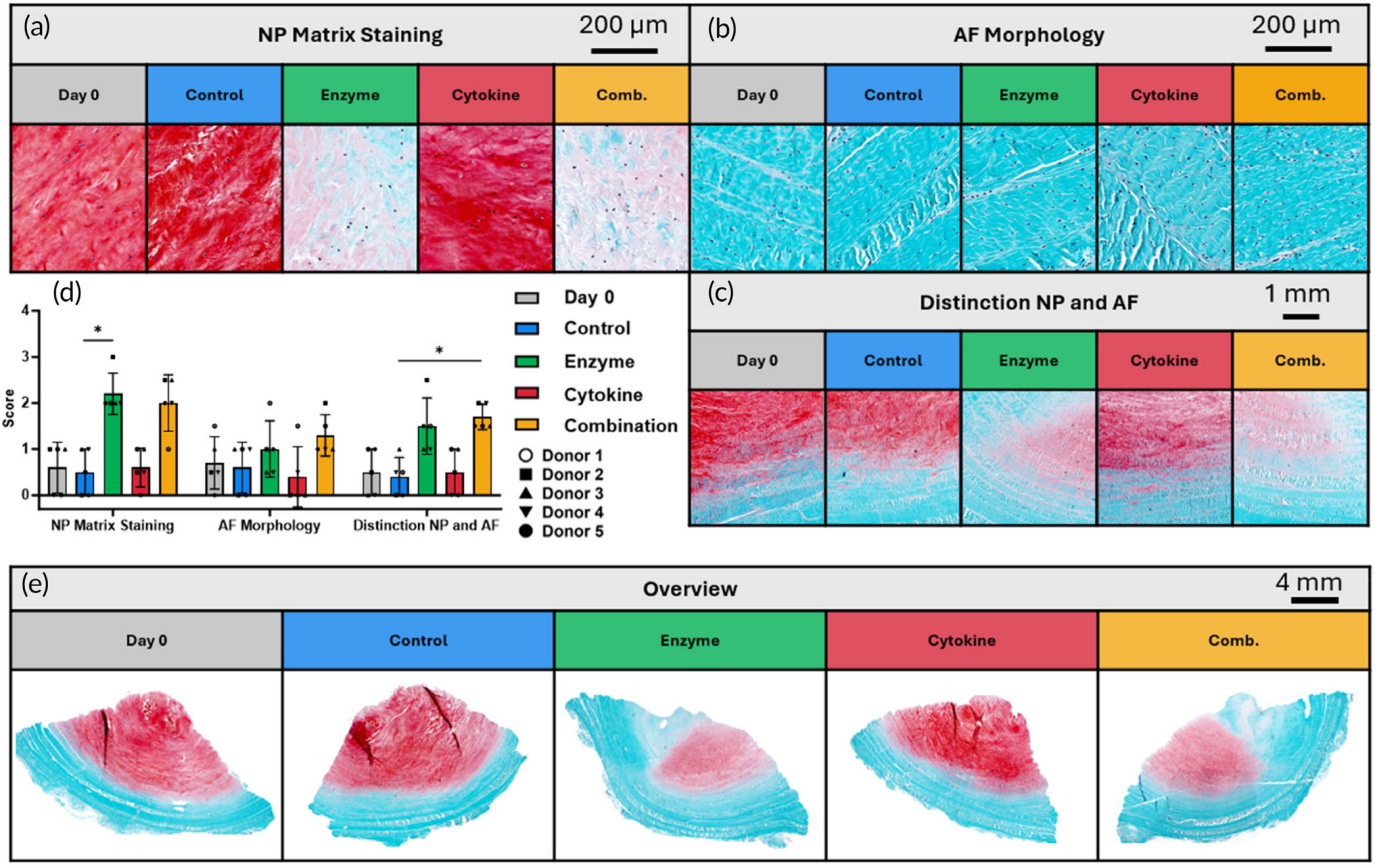
Safranin O/Fast Green staining and histological degeneration score. (a–c) Representative images for NP matrix staining, AF morphology and Distinction NP and AF. (d) Histological degeneration score, performed by two blinded researchers. Shown for each datapoint is the average of the individual scores. Kruskal–Wallis with Dunn's multiple comparisons test was used to compare groups; *n* = 5 and *p*‐values <0.05 were considered as statistically significant (*p < 0.05, **p < 0.01, ***p < 0.001). (e) Representative images of whole sections for all experimental groups.

For the enzyme and combination groups, Safranin O/Fast Green‐stained sections showed a noticeable reduction in proteoglycan staining (red) in the NP (Figure 4a). This was reflected by a significant 3.7‐fold increase of the degenerative score for NP matrix staining in the enzyme group compared to the control group (Figure 4d). AF morphology remained intact across all groups, with a trend toward mild disorganization in the two papain‐injected groups (Figure 4b, d). For NP‐AF distinction, the papain‐injected groups exhibited higher degeneration scores, and a significant 4.25‐fold increase was observed in the combination group compared to the control group (Figure 4d, c). This suggests a loss of annular‐nuclear demarcation for the enzyme and combination groups.

### 3.4 Biomechanical parameters

**FIGURE 5:**
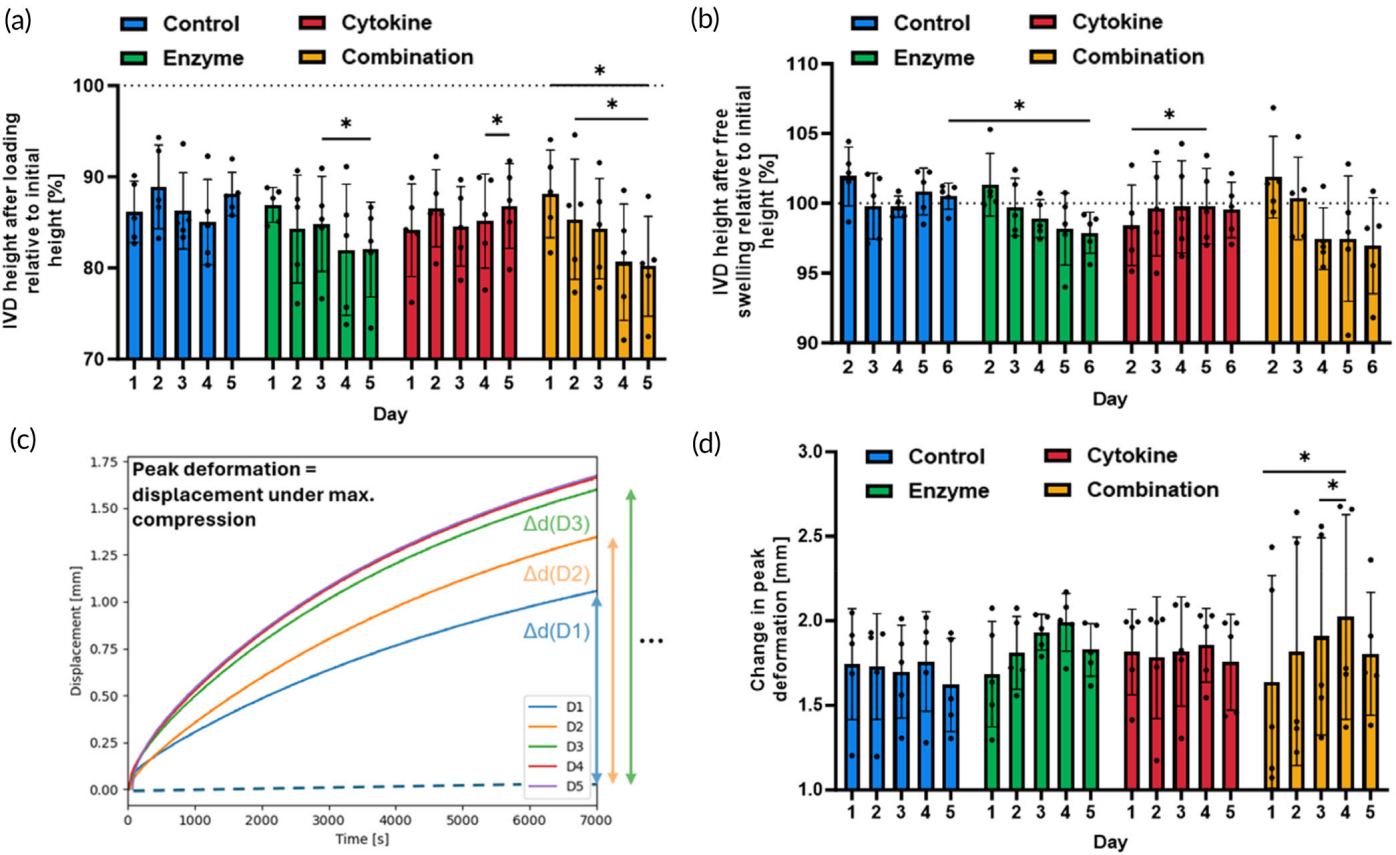
Biomechanical characterization. (a) IVD height after loading relative to initial height for all five loading days. (b) IVD height after free swelling relative to initial height for all five loading days. (c) Representative graph for peak deformation for one exemplary sample and all loading days. Change in peak deformation (Δ*d*) was defined as the displacement under maximal compression on the last cycle minus the displacement under maximal compression on the first cycle for every loading day (D1–D5). (d) Change in peak deformation for the different groups and days. For all datasets, *n* = 5 IVDs, and repeated measure two‐way ANOVA with Geisser Greenhouse correction and Tukey's multiple comparisons test were used to compare groups and time effects for all biomechanical datasets. *p*‐values <0.05 were considered statistically significant (*p < 0.05).

IVD height after loading decreased significantly from 88.15% and 85.35% to 80.19% on day 5 compared to day 1 and 2 for the combination group (Figure 5a). Moreover, a significant decrease was observed in the enzyme group on day 5 compared to day 3. In the cytokine group, a significant difference was found between days 4 and 5. Looking at IVD height after free swelling, all groups were on average able to recover the initial IVD height ±5% (Figure 5b). IVD height after free swelling was significantly lower in the enzyme group than in the control group on day 6. A similar trend was observed for the combination group, despite not being statistically significant. In addition, a statistical difference was found within the cytokine group between days 2 and 5. Overall loading accuracy was high with an average mean absolute error of 0.97 ± 0.42 N and 0.92 ± 0.52 N for average max and min target forces of 50.3 and 5.04 N, respectively. Change in peak deformation was calculated as peak deformation at the last cycle minus peak deformation at the first cycle (Figure 5c). The combination group showed a significant increase in maximal peak deformation between days 1 and 4 as well as between days 3 and 4 (Figure 5d). A similar trend could be seen for the enzyme group; however, the increase was not significant. For the control group and the cytokine group, peak deformation stayed relatively constant with standard deviations over the 5 days of loading of ±0.055 and 0.042 mm, respectively.

### 3.5 Inflammatory cytokine release and IVD cell gene expression

**FIGURE 6:**
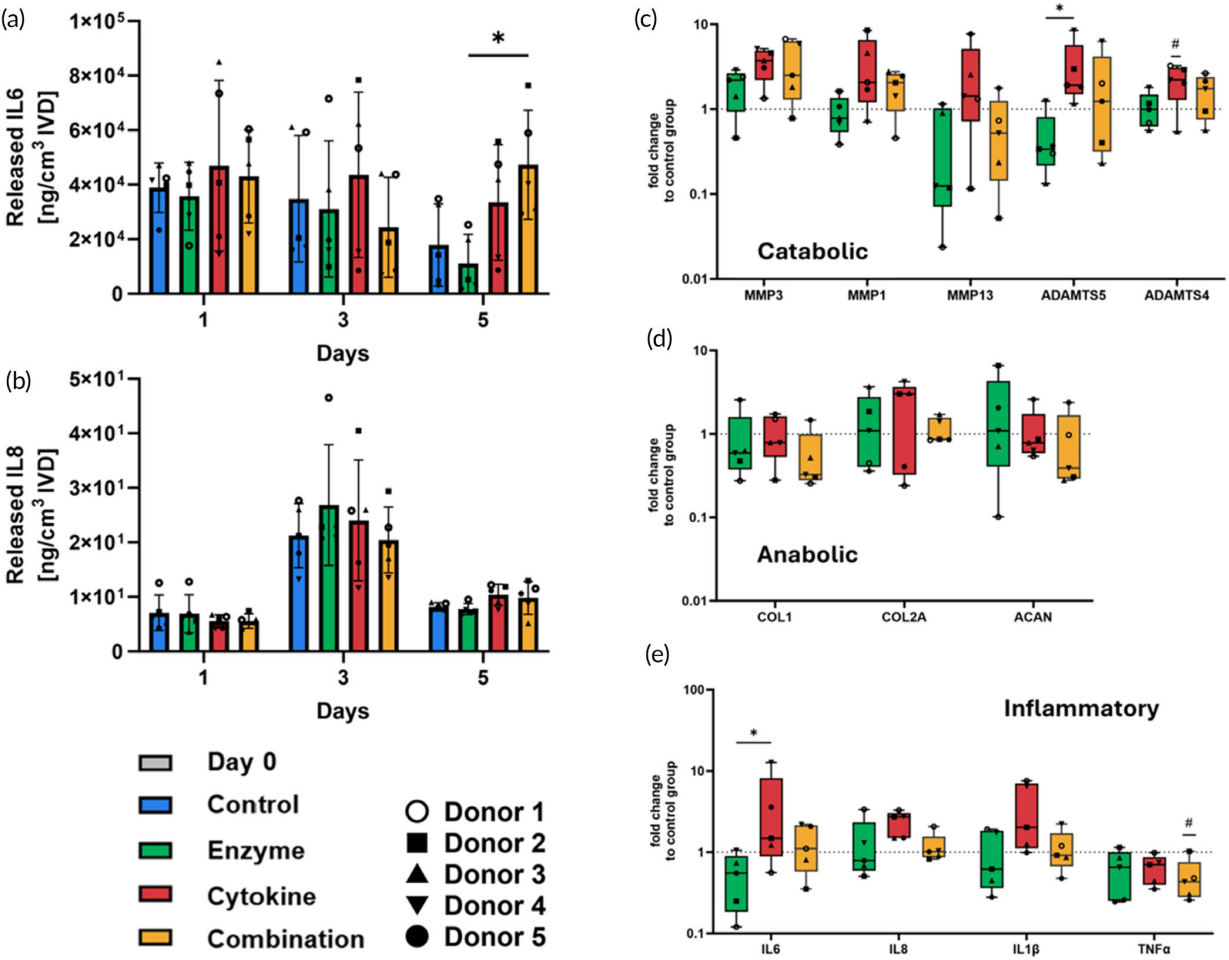
Cytokine release (a, b) and gene expression of IVD cells (c–e). (a) IL6 release on days 1, 3 and 5. (b) IL8 release on days 1, 3 and 5. For all ELISA data, groups were compared for each day individually using a one‐way ANOVA with Tukey's multiple comparisons test or Kruskal–Wallis with Dunn's multiple comparisons test. Catabolic (c), anabolic (d), and inflammatory (e) genes measured using RT‐qPCR showed on a log scale. The boxplot shows the median and min to max range relative to the reference gene and the control group (*n* = 5). Groups were compared for every gene by either using one‐way ANOVA with Dunnett's multiple comparisons test and Tukey's multiple comparisons test or Kruskal–Wallis with Dunn's multiple comparisons test. To also assess differences to the reference group a Bartlett's test was performed for all genes. P‐values <0.05 were considered as statistically significant (*/#*p* < 0.05). Asterisks (*) annotate significances among treatments, whereas dashes (#) annotate significant differences to the reference group (control group).

On days 1 and 3, the IL6 release showed no significant difference among the experimental groups. On day 5, IL6 content in medium was 4.27 times higher for the combination group compared to the enzyme group (Figure 6a). Despite no significant differences between the cytokine or combination groups and the control group, IL6 release was still 1.87 and 2.65 times higher, respectively. No difference in IL8 release was observed among the treatment groups (Figure 6b).

At the gene expression level, catabolic genes (matrix metalloproteinases [MMP]: MMP1, MMP3, MMP13 and a disintegrin and metalloproteinase with thrombospondin motifs [ADAMTS]: ADAMTS4 and ADAMTS5) were upregulated in the cytokine group relative to the control group; the expression of ADAMTS5 was significantly (6.85‐fold) higher in the cytokine compared to the enzyme group, and ADAMTS4 was significantly (2.19‐fold) higher in the cytokine than the control group (Figure 6c). Anabolic genes (collagen [COL]: COL1, COL2A, and aggrecan [ACAN]) had variable expression with no significant group differences (Figure 6d). The inflammatory markers IL6, IL8, and IL1β showed a trend of upregulation in the cytokine group and downregulation in the enzyme group, while the combination group remained close to the control (Figure 6e). IL6 expression was 7.20‐fold higher in the cytokine compared to the enzyme group, resulting in a significant difference. TNFα was downregulated in all groups and significantly downregulated in the combination compared to the control (reference) group.

### 3.6 Effect on neural sensitization of IVD CM treatment

**FIGURE 7:**
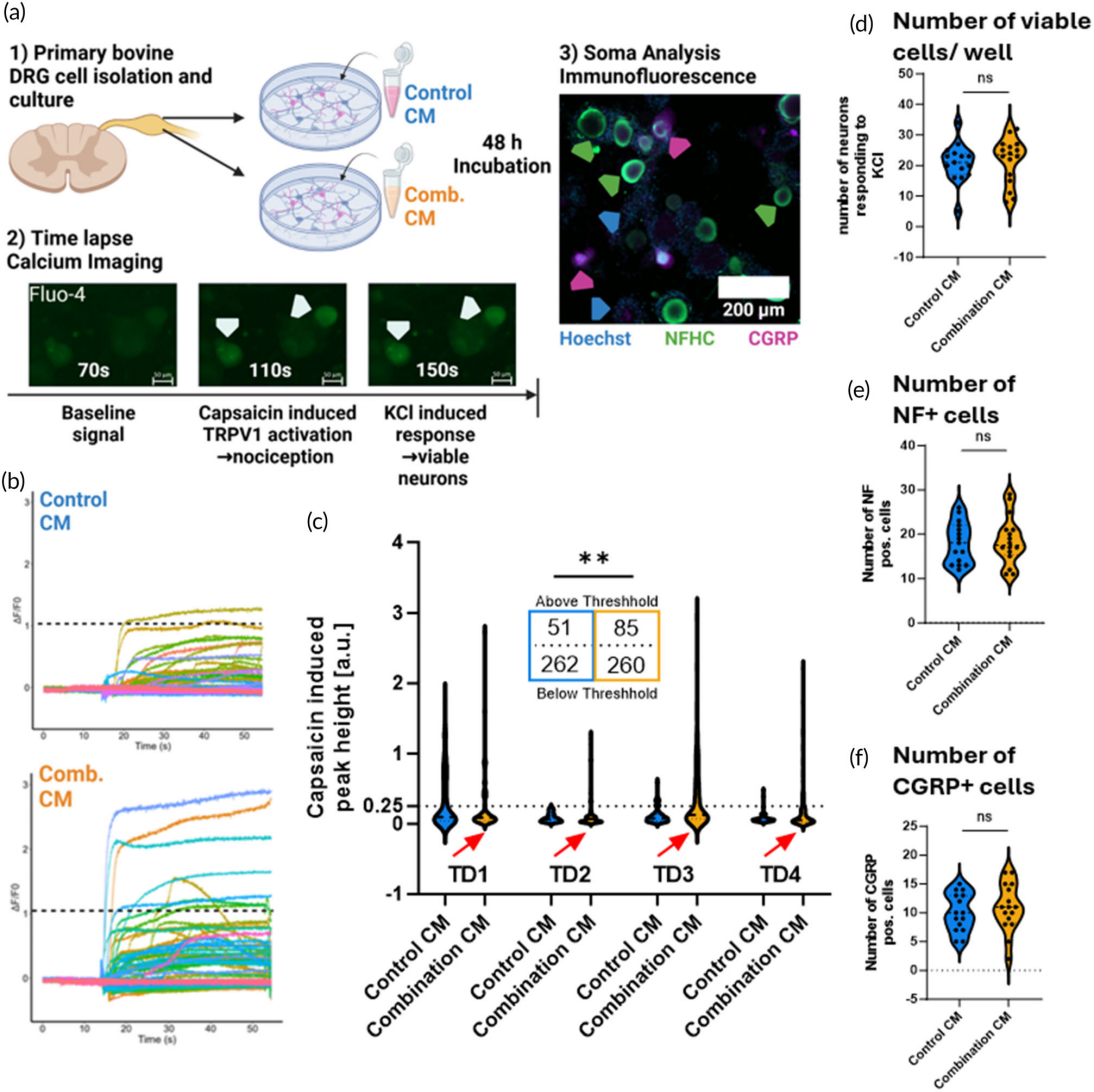
(a) Overview of the experimental setup using IVD‐CM from the combination and control groups (*n* = 4 tail donors [TDs] and *n* = 4 DRG donors [DDs]). Data are shown as violin plots. (b) Normalized change in fluorescence for one representative DD for all measured TDs (F0 = baseline fluorescence, ΔF = difference to baseline). The x‐axis is cut to not have full baseline measurement visible but only the last 55 s of the measurement. Dotted line was added for easier comparisons of the two plots. (c) Neural sensitization measured by calcium imaging. The threshold to classify neurons for statistical analysis was set to 0.25 and a chi‐square test was used to evaluate statistical differences. A graph showing individual datapoints can be found in Data S6. (d) Absolute number of cells per well that reacted to KCl stimulation as an indicator of viability. (e) Absolute number of NF+ cells per well. (f) Absolute number of CGRP+ cells per well. For (d–f) statistical test used was either an unpaired *t* test or a Mann–Whitney U test. *p*‐values <0.05 were considered as significant (**p < 0.01).

To test the nociceptive effect of released factors accumulated in CM, primary DRG neurons were stimulated for 48 h with CM from the combination or the control group, followed by calcium imaging and immunofluorescence (Figure 7a). Overall, 1.51 times more neurons exhibited a capsaicin‐induced response exceeding the 0.25 threshold when exposed to combination group CM, compared to control group CM (Figure 7c). Specifically, 24.6% of neurons in the combination group and 16.3% of neurons in the control group surpassed the threshold, resulting in a significant difference. This is also apparent qualitatively by looking at the normalized fluorescence of the detected neurons over time (Figure 7b, shown for one DRG donor).

The number of viable neurons was not significantly different between the two groups (Figure 7d). Immunofluorescence imaging showed no differences in the number of NF+ cells and only marginal differences in CGRP+ cells between the combination and control groups (Figure 7e, f).

TNFα content within the CM used in the DRG sensitization study was measured with ELISA. Whereas the levels of the control group remained below the detection limit of 125 pg/mL, the combinatory group showed an average TNFα content of 0.82 ± 0.34 ng/mL (Data S8).

## 4 DISCUSSION

This study aimed to establish a novel multifactorial disease model of IVDD to enable testing of complex treatment strategies. Toward this aim, the combined effects of previously used disease inducers (papain and TNFα) on bovine IVDs were examined.

Importantly, papain injection did not affect cell viability, which is consistent with the results reported by Vernengo *et al*., even though they used a 40× higher dose of papain and, due to larger void formation, could only analyze cell viability in the AF.^23^ Although cell death is a hallmark of IVDD, maintaining partial cell viability in organ culture models is essential to assess cellular responses to treatments tested.

Papain injection resulted in a void in the NP that was visible at a macroscopic level and will enable the application of intradiscal therapies. As shown in previous studies, papain indeed is capable of IVD ECM breakdown by enzymatic cleavage of linker and core proteins of aggrecan leading to a measurable GAG loss.^23^, ^37^, ^45^ GAG loss was shown by reduced NP GAG content and increased GAG release in the medium. Confirming the assumption that GAG loss mainly originated from NP, histological scoring showed that NP and NP‐AF transition zone were mostly affected, whereas AF remained intact.

The progressive papain‐induced matrix breakdown was associated with a decrease in height after loading and an increase in peak deformation, with slightly greater changes in the combination group. This further suggests that enzymatic degradation was the primary driver of ECM breakdown, while TNFα may have contributed modestly to the observed effects. Another hallmark of IVDD is a compromised swelling capacity. In our model, swelling tended to diminish after enzyme injection, suggesting a decrease in reswelling capacity.

Despite the decrease in NP GAG content and reswelling capacity, tissue hydration in the papain group was only 1.04% and 1.38% lower than the control and the day 0 group, respectively. This suggests that the remaining IVD ECM components compensated for the loss of NP GAGs during the overnight free swelling period, allowing the IVD to maintain its water content. A valuable follow‐up experiment would be to measure hydration immediately after the final loading period and at several timepoints thereafter, to investigate whether the reduction in NP GAGs influences the rehydration rate post‐loading. Notably, control oAF showed a significant increase in hydration compared to day 0 and the two enzyme groups, while the cytokine group followed a similar trend, with hydration levels higher than those of day 0 samples. In vivo, the outer oAF border is surrounded by connective tissue, muscles, and ligaments, which constrain swelling and water uptake due to the well‐defined, pressurized environment. However, this boundary is lost in IVD organ cultures, where the oAF directly interfaces with the liquid culture medium. We therefore hypothesize that the increased hydration observed in the control group is an artifact of the IVD organ culture system. Interestingly, this effect is absent in the papain‐treated groups, where oAF hydration remains similar to day 0. Further investigation is needed to elucidate the underlying mechanisms. Advanced MRI techniques that distinguish between bound and unbound water (e.g., T2 relaxometry or chemical exchange saturation transfer techniques) could help determine whether and where the tissue's water‐binding capacity is compromised, offering a more specific and physiologically relevant parameter than total hydration alone.

Different from papain, TNFα was not found to damage the ECM structurally. As expected, TNFα injection upregulated the release and gene expression of IL6. The combination of TNFα and papain showed a trend of a lower IL6 and IL8 gene expression compared to TNFα injection alone. In contrast, IL6 release at the protein level was higher in the combination group compared to TNFα alone. We hypothesize that papain injection may have facilitated IL6 release due to a loosening of ECM components, thereby contributing to the observed increase of IL6 in the medium.

Interestingly we did not find any significant difference in IL8 expression or release after cytokine treatment, despite previous studies showing an increase in IL8 release following TNFα injection at similar concentrations.^25^ The different outcome might be related to the different timepoints chosen for cytokine administration (day 1 vs. day 3; analysis for both on day 5). This highlights the importance of choosing appropriate timepoints when studying inflammation, as inflammatory markers can fluctuate rapidly over time. Many other genes associated with IVDD and inflammation (e.g., ADAMTS5, MMP1, ADAMTS4, IL6) were found to be only marginally affected by TNFα injection. One limitation of our study was that tissue for RNA isolation was collected using cryosections, which do not allow for clear separation of the AF and NP. This may have masked AF‐ and NP‐specific effects in our analyses and led to the partly marginal differences. Another explanation could be that TNFα stimulation may only be effective for a short time, and other cytokines involved in IVDD progression were not included in our model. To achieve greater cytokine effects, using higher TNFα concentrations or repeated injections could result in a more pronounced inflammatory and catabolic shift. Alternatively, combining multiple cytokines, as previously demonstrated with TNFα and IL1β, could provide a more comprehensive means of inducing an inflammatory and catabolic IVD phenotype.^46^ In addition, emerging omics approaches, including proteomics and transcriptomics, may reveal differences that the current targeted analyses overlooked.

An important limitation of this study is the low sample size increasing the chance of random variation. Nevertheless, clear and meaningful differences between experimental groups were observed. Furthermore, such sample sizes are typical in this field due to the labor‐intensive and technically demanding nature of these experiments. If higher‐throughput experiments are required, recently developed organ‐on‐a‐chip IVD models or 3D‐printed IVD constructs may offer more suitable alternatives.^47^, ^48^, ^49^

The second goal of this study was to incorporate an *in vitro* experiment to evaluate nociceptive components within the workflow of whole organ experiments. A similar workflow has previously been used by Gewiess *et al*. who showed that factors released by overloaded IVDs increased nociceptor flickering.^34^ The focus of this study was placed on the combinatory disease model and the question of whether it could be used to assess a treatment's effect on nociceptive outcomes. Therefore, CM from only the combinatory and the control groups were used. We believe that our results primarily reflect the downstream effects induced by injected papain and TNFα, rather than their direct actions. Given the short half‐life of papain, it was likely inactivated by the time of CM collection. The measured TNFα concentration in the CM, including potential residual injectate and any TNFα produced by IVD cells, was 0.82 ng/mL. Although TNFα also has a known short half‐life, for example in serum, we cannot entirely exclude any direct influence of either injectate on the CM.^50^ Our results show a significantly higher number of neurons responding to nociceptive capsaicin stimuli after exposure to combination CM compared to control CM. Capsaicin is the agonist of the transient receptor potential cation channel subfamily V member 1 (TRPV1) which is a key regulator in the sensation of heat and pain.^51^ Our data suggest that the combination CM lowered the nociceptive threshold, indicating sensitization of nociceptive neurons compared to the control group. Testing a therapy that can reverse or reduce this pre‐sensitization may provide valuable insight into its efficacy in modulating nociceptive signaling. Interestingly, we did not observe any differences in CGRP and NF expression or viability between the groups. Potentially, longer exposure to IVDD CM may cause a change of CGRP level and non‐nociceptor/nociceptor phenotype switch. Here, we only compared the combination and the control group. The sole effect of either TNFα or papain was not assessed and still needs to be evaluated in the future. Furthermore, it is important to study the molecular mechanism behind IVD‐DRG communication and nociceptor activation. Both ECM components and inflammatory molecules (such as IL6 and IL8) have been shown to have signaling roles in IVD‐DRG crosstalk.^52^, ^53^, ^54^, ^55^, ^56^ Intact GAGs have been associated with limiting neuronal ingrowth into healthy IVDs—an effect lost once GAGs are enzymatically cleaved.^57^, ^58^ It is possible that GAG fragments are capable of increasing neuron sensitization, though further experiments are needed to validate this hypothesis.

Overall, in this combinatory model, structural alterations and nociceptive sensitization are evident, whereas inflammatory and catabolic responses remain relatively moderate. This pattern suggests that the model most closely reflects an early‐ to mid‐stage IVDD. Notably, such a stage would represent an optimal window for evaluating regenerative interventions, such as cell‐based therapies.^28^

## 5 CONCLUSIONS

We developed an IVDD model combining papain and TNFα to replicate the structural breakdown, release of inflammatory mediators, and catabolic cellular shift. Compared to single degeneration inducers, this approach more accurately reflects the complex, multifactorial nature of IVDD, enhancing the robustness of testing treatment strategies like cell therapies. Incorporating nociceptive outcomes into IVD organ model experiments improves insights into the potential pain alleviation of regenerative treatments. While these biological assessments of neurons cannot directly represent pain assessment, the proposed model can serve as a valuable complement to, or even replacement for, *in vivo* animal models, advancing the “Replacement” aspect of the 3Rs principles.

## AUTHOR CONTRIBUTIONS

Marcia Muerner: conceptualization, methodology, validation, formal analysis, investigation, visualization, writing – original draft; Junxuan Ma: methodology, formal analysis, investigation, visualization, writing – original draft, writing – review and editing; Rathina V. Balasubramanian: conceptualization, methodology, writing – review and editing; Chencheng Feng: methodology, investigation, writing – review and editing; Julia Fernández Pérez: conceptualization, project administration, writing – review and editing; Aleksandr Ovsianikov: conceptualization, resources, funding acquisition, project administration, writing – review and editing; Sibylle Grad: conceptualization, methodology, resources, funding acquisition, project administration, writing – review and editing.

## CONFLICT OF INTEREST STATEMENT

All authors declare that they have no conflicts of interest with respect to this work.

## ETHICS STATEMENT

All tissues used for this study were obtained from animals sacrificed for meat production. No ethical approval was required to carry out the experiments.

## Supporting information


**Data S1:** In vitro feasibility study: combining papain and TNFα.
**Data S2:** Comparison of manually counted versus automatically counted viability.
**Data S3:** Sequences used for RT‐qPCR.
**Data S4:** Representative images of cell viability assessment using LDH/EtH staining.
**Data S5:** Cumulative glycosaminoglycan release graph with all datapoints.
**Data S6:** Neural sensitization measured by calcium imaging.
**Data S7:** Pilot ELISA results following TNFα injection into bovine IVDs (dose: 250 ng, injected on day 1 post‐loading) showed peak IL6 and IL8 release between days 2 and 3 (*N* = 2 IVDs). Some IL6 concentrations exceeded the assay's linear range; therefore, absolute values should be interpreted with caution.
**Data S8:** TNFα content of the conditioned medium (CM) used for neuron sensitization. Whereas the control medium had a concentration below the limit of detection (125 pg/mL), the combinatory group showed an average TNFα content of 0.82 ± 0.34 ng/mL (*n* = 4).

## Data Availability

The data that support the findings of this study are available from the corresponding author upon reasonable request.
