# Supplementary material for: A multifactorial intervertebral disc degeneration model: Integrating inflammation, structural disruption, biomechanical parameters, and neural sensitization
Source: Bioeng Transl Med. 2026 Feb 1;11(3):e70120. doi: 10.1002/btm2.70120 (PMC13247399; doi:10.1002/btm2.70120)
Supplement: Supplementary file 1 — Data S1: In vitro feasibility study: combining papain and TNFα. Data S2: Comparison of manually counted versus automatically counted viability. Data S3: Sequences used for RT‐qPCR. Data S4: Representative images of cell viability assessment using LDH/EtH staining. Data S5: Cumulative glycosaminoglycan release graph with all datapoints. Data S6: Neural sensitization measured by calcium imaging. Data S7: Pilot ELISA results following TNFα injection into bovine IVDs (dose: 250 ng, injected on day 1 post‐loading) showed peak IL6 and IL8 release between days 2 and 3 (N = 2 IVDs). Some IL6 concentrations exceeded the assay's linear range; therefore, absolute values should be interpreted with caution. Data S8: TNFα content of the conditioned medium (CM) used for neuron sensitization. Whereas the control medium had a concentration below the limit of detection (125 pg/mL), the combinatory group showed an average TNFα content of 0.82 ± 0.34 ng/mL (n = 4). [file BTM2-11-e70120-s001.docx]

**Supplementary Information:**

**A Multifactorial Intervertebral Disc Degeneration Model: Integrating Structural Disruption, Inflammation, Biomechanical Parameters, and Neural Sensitization**

S1: *In vitro* feasibility study: Combining papain and TNFα.

To test compatibility of papain and TNFα, the two reagents were mixed and incubated at 20% oxygen and 37°C at a final reagent activity/ concentration of 10 ng/mL for TNFα and 6 U/mL for papain. To enable optimal enzyme activity, papain (Merck, Switzerland) was dissolved at an initial activity of 60 U/mL in a PBS buffer containing 5 mM ethylenediaminetetraacetic acid (EDTA, MW 372.24) and 10 mM L-cysteine HCl (MW 157.62) as described in (1). The papain buffer was adjusted to a pH of 6.4 and sterile filtered. Recombinant bovine TNFα (R&D biotech, France) was dissolved in PBS. After 2 hours, 1, 3 and 7 days, 0.5 mL of each mix was harvested (n=4). TNFα content in the different groups was measured with an enzyme-linked immunosorbent assay (ELISA, R&D systems, USA) using the manufacturer’s instructions. Groups with TNFα additions were tested for all harvested timepoints, whereas groups without TNFα addition where only tested after 2 hours and 7 days as negative controls.

When papain was present in the mix, TNFα was not detectable anymore via ELISA. Therefore, sequential injection of the two reagents seemed more promising and was implemented in the model.

(1)Gantenbein, B., et al., *Organ culture bioreactors--platforms to study human intervertebral disc degeneration and regenerative therapy.* Curr Stem Cell Res Ther, 2015. **10**(4): p. 339-52.

S2: Comparison of manually counted vs automatically counted viability. For cell viabilities between 70-100% a strong correlation was observed. N=9, from NP, iAF and oAF regions.

S3: *Sequences of primers and probes used for RT-qPCR.*

| Gene Name | Forward  Or  Assay ID if commercial | Reverse | Probe | Probe 5' Modification/ 3' Modification |
| --- | --- | --- | --- | --- |
| Primer-Probe designed (Microsynth, Switzerland) | | | | |
| Aggrecan (ACAN) | 5'-CCA ACG AAA CCT ATG ACG TGT ACT-3' | 5'-GCA CTC GTT GGC TGC CTC-3' | 5'-ATG TTG CAT AGA AGA CCT CGC CCT CCA T-3' | FAM/TAMRA |
| A disintegrin and metalloproteinase with thrombospondin motifs (ADAMTS) 4 | 5'-CCC CAT GTG CAA CGT CAA G-3' | 5'-AGT CTC CAC AAA TCT GCT CAG TGA-3' | 5'-AGC CCC CGA AGG GCT AAG CGC-3' | FAM/TAMRA |
| ADAMTS5 | 5'-GAT GGT CAC GGT AAC TGT TTG CT-3' | 5'-GCC GGG ACA CAC CGA GTA C-3' | 5'-AGG CCA GAC CTA CGA TGC CAG CC-3' | FAM/TAMRA |
| Collagen (COL) -1 | 5'-TGC AGT AAC TTC GTG CCT AGC A-3' | 5'-CGC GTG GTC CTC TAT CTC CA-3' | 5'-CAT GCC AAT CCT TAC AAG AGG CAA CTG C-3' | FAM/TAMRA |
| COL2A | 5'-AAG AAA CAC ATC TGG TTT GGA GAA A-3' | 5'-TGG GAG CCA GGT TGT CAT C-3' | 5'-CAA CGG TGG CTT CCA CTT CAG CTA TGG-3' | FAM/TAMRA |
| Matrix Metallopeptidase (MMP) 1 | 5'-TTC AGC TTT CTC AGG ACG ACA TT-3' | 5'-CGA CTG GCT GAG TGG GAT TT-3' | 5'-TCC AGG CCA TCT ACG GAC CTT CCC-3' | FAM/ TAMRA |
| MMP3 | 5'-GGC TGC AAG GGA CAA GGA A-3' | 5'-CAA ACT GTT TCG TAT CCT TTG CAA-3' | 5'-CAC CAT GGA GCT TGT TCA GCA ATA TCT AGA AAA C-3' | FAM/ TAMRA |
| MMP13 | 5'-CCA TCT ACA CCT ACA CTG GCA AAA G-3' | 5'-GTC TGG CGT TTT GGG ATG TT-3' | 5'-TCT CTC TAT GGT CCA GGA GAT GAA GAC CCC-3' | FAM/ TAMRA |
| Interleukin (IL) 1β | 5'-TTA CTA CAG TGA CGA GAA TGA GCT GTT-3' | 5'-GGT CCA GGT GTT GGA TGC A-3' | 5'-CTC TTC ATC TGT TTA GGG TCA TCA GCC TCA A-3' | FAM/ TAMRA |
| IL6 | 5'-TTC CAA AAA TGG AGG AAA AGG A-3' | 5'-TCC AGA AGA CCA GCA GTG GTT-3' | 5'CTT CCA ATC TGG GTT CAA TCA GGC GAT T-3' | FAM/ TAMRA |
| Commercially purchased primer mix (Thermofisher, USA) | | | | |
| IL8 | Bt03211906_m1 | - | - | FAM/NFQ |
| RPLP0 | Bt03218086_m1 | - | - | FAM/NFQ |

S4: Representative Images of LDH / EtH staining used to assess cell viability


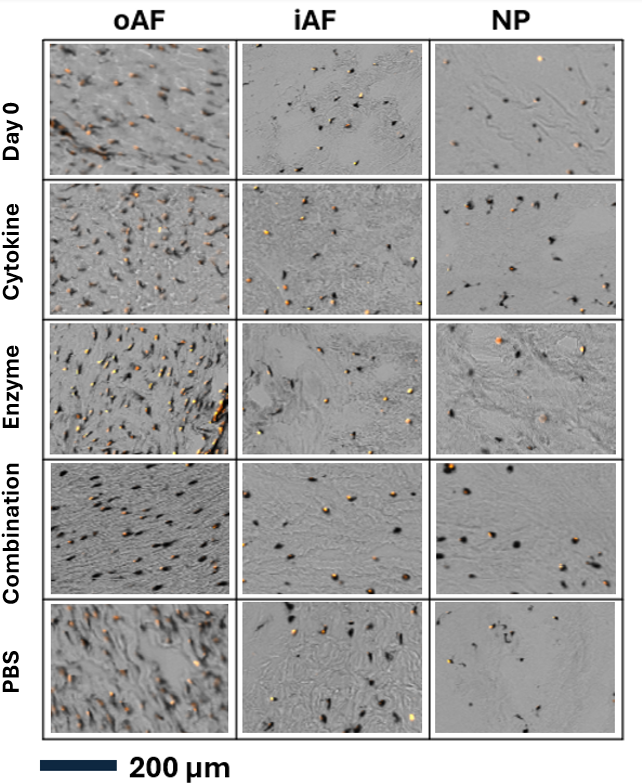


S5: Cumulative glycosaminoglycan release graph with all datapoints. The same data as Figure 3A of the main manuscript are shown but displayed as individual datapoints instead of mean values.

S6: Neural sensitization measured by calcium imaging. The same data as Figure 7C of the main manuscript are shown but displayed as individual datapoints instead of a violin plot.

S7: Pilot ELISA results following TNFα injection into bovine IVDs (dose: 250 ng, injected on day 1 post-loading) showed peak IL-6 and IL-8 release between days 2–3 (N = 2 IVDs). Some IL-6 concentrations exceeded the assay’s linear range; therefore, absolute values should be interpreted with caution.


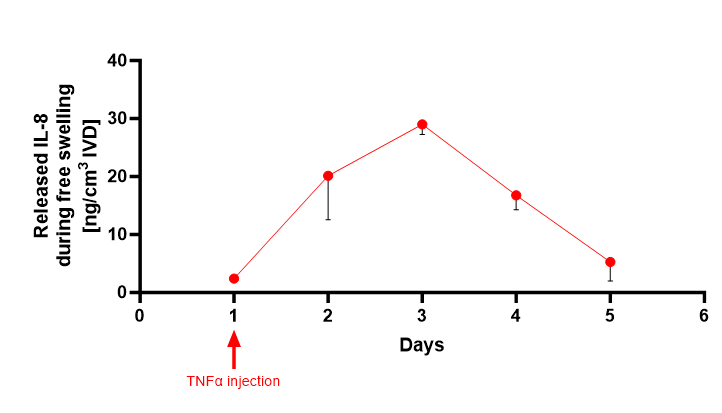


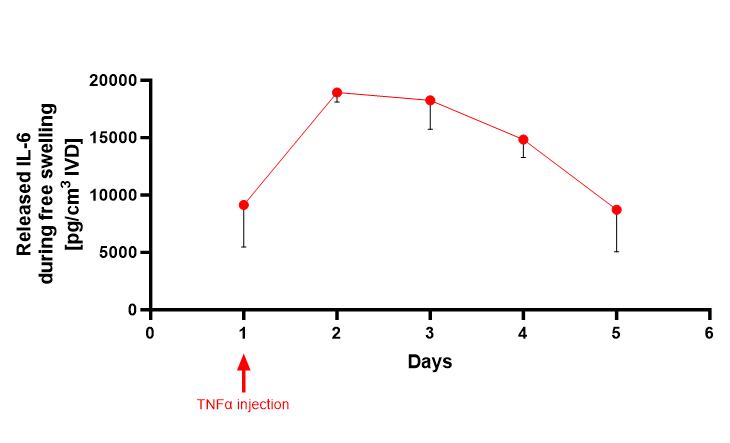


S8: TNFα content of the conditioned medium (CM) used for neuron sensitization. Whereas the control medium had a concentration below the limit of detection (125 pg/mL), the combinatory group showed an average TNFα content of 0.82 ± 0.34 ng/mL (n=4).

Initial dose of TNFα injectate: 250 ng

Total measured TNFα in the medium:
5 mL (medium volume) * 0.82 ng/mL (average concentration) = 4.1 ng
